# Naturally Occurring Compounds/Materials as Alternatives to Synthetic Chemical Insecticides for Use in Fire Ant Management

**DOI:** 10.3390/insects11110758

**Published:** 2020-11-04

**Authors:** Jian Chen, David H. Oi

**Affiliations:** 1 National Biological Control Laboratory, Southeast Area, Agriculture Research Service, United States Department of Agriculture, 59 Lee Road, Stoneville, MS 38776, USA; 2 Center for Medical, Agricultural, and Veterinary Entomology, Southeast Area, Agriculture Research Service, United States Department of Agriculture, 1600 SW 23rd Drive, Gainesville, FL 32608, USA; david.oi@usda.gov

**Keywords:** red imported fire ant, active ingredient, bait, mound treatment, repellant, fumigant, natural products

## Abstract

**Simple Summary:**

Red imported fire ants are a notorious pest, impacting humans, livestock, pets and wildlife due to their venomous stings and causing billions of dollars in damages annually. Synthetic insecticides are a major tool used to control this pest. There is an ever-increasing public concern about the potential adverse effects of synthetic insecticides. Extensive effort has been made in searching for alternatives. In addition to biological control, physical and cultural practices, and semiochemicals, natural products continue to be one of the most attractive sources of alternatives. Naturally occurring compounds/materials have been successfully used as active ingredients in fire ant baits, contact-based control products, repellants and fumigants. In this article, we summarized the synthetic insecticides that are currently used in managing fire ants, available alternative products in the current USA market, and academic efforts in searching for fire ant natural toxins, repellants and fumigants.

**Abstract:**

The invasive red imported fire ant, *Solenopsis invicta* Buren (hereafter, fire ants), is a significant threat to public health and a danger to livestock, pets and wildlife due to their venomous stings. The fire ant has invaded many countries and regions and has become a globally significant pest. The current major tool to manage fire ants are synthetic insecticides that are used largely as stomach poisons in bait products or contact insecticides in spray, broadcast, drench, and dust products for area and nest treatments. In addition to these insecticide products, repellants and fumigants can also be useful in some unique scenarios. The ever-increasing public concern about the potential adverse effects of synthetic insecticides on health and the environment has been a driving force for searching for safer alternatives to control fire ants. Tremendous effort has been made in developing biologically-based control for managing fire ants; however, natural products continue to be one of the most attractive sources of safe alternatives to synthetic insecticides. Here, we summarized the synthetic insecticides that are currently used in managing fire ants, available alternative products in the current market, and academic efforts in searching for fire ant natural toxins, repellants and fumigants.

## 1. Introduction

Red imported fire ants, *Solenopsis invicta* Buren (hereafter, fire ants), are a significant threat to public health and a danger to livestock, pets and wildlife due to their venomous stings. Fire ants are also a significant agricultural pest because they can damage many crops [1]. As one of the worst invasive species, fire ants, which are originally from South America, have been introduced into many countries and regions and have become an important global pest [2]. Synthetic insecticides continue to be a common tool for managing fire ants. Some of synthetic insecticides are structurally adapted from natural toxins; however, the modified synthetic versions were often preferred by manufacturers for several possible reasons including (1) natural toxins are often not suitable to be directly used as an insecticide due to their physiochemical properties, (2) structural complexity of the natural toxins often makes their synthesis economically infeasible, (3) alteration of natural toxins often improve their efficacy, and (4) intellectual property rights of synthetic insecticides are easier to obtain and defend than the natural toxins [3]. However, due to the ever-increasing concern over the negative impact of synthetic insecticides on public health and the environment, there is a growing desire to use more toxicological benign and environmentally friendly products and methods in pest management. Fire ant management is no exception. In addition to biological control [4], physical and cultural practices [5,6,7], and semiochemicals [8,9], natural products continue to be one of the most attractive sources of alternatives.

The National Pesticide Information Center defines insecticides as pesticides that are formulated to kill, harm, repel or mitigate one or more species of insect (http://npic.orst.edu/ingred/ptype/insecticide.html). Based on these criteria, four types of insecticides are used in fire ant management, including slow-acting stomach poisons which contain metabolic inhibitors or insect growth regulators, fast-acting contact poisons, repellants, and fumigants. Slow-acting stomach poisons are used in bait products and contact poisons in spray, broadcast, drench, or dust formulations for area and direct nest treatment. Repellants can be useful to exclude ants from some sensitive areas, such as electrical equipment and in hospitals and schools, where synthetic insecticides may be not desired. Fumigants can be useful not only in nest treatment, but also potentially in quarantine treatments, such as disinfesting fire ants from commercial shipping containers. Here we review the current status of chemical control in fire ants, available alternatives to synthetic insecticides, and compounds/materials that recently have been tested for their toxicity and repellency against fire ants.

## 2. Alternatives to Synthetic Chemical Insecticides for Use in Fire Ant Bait Products

### 2.1. Overview

The collective foraging behavior of ants provides a good opportunity to utilize bait technology in their management. Foraging workers find and transfer bait back to the colony by trophallaxis, which allows the active ingredient (AI) contained in baits to be spread to other ants within their colony. When the AI is distributed to most of the colony members the ant colony will die. Baits are easy to apply and are environmentally friendly and safer to use because they require significantly less AI compared to other chemical control methods. Consequently, baits have become a preferred method in managing many pest ants, including imported fire ants. AIs need to be slow-acting because foraging workers need enough time to spread the AI within a colony before the onset of the toxic effect. The requirement of a slow-acting AI has significantly limited the number of insecticides that can be utilized as ant bait AIs. In fact, there are only eight AIs in commercial fire ant baits in the current US market (Table 1).

Such requirement also renders most ant bait products to have relatively slow (≥2 weeks) impact on ant colonies when compared to most contact-based products which can affect colonies in minutes to days. However, faster acting baits have been developed. Baits containing indoxacarb and metaflumizone can kill colonies in a week or less [10,11]. The slow-acting nature of most ant baits can be problem when a quick control is needed.

Fire ant bait AIs are dominated by synthetic insecticides. Among eight AIs (Table 1), six are synthetic insecticides. The search for synthetic bait AIs has continued. Two synthetic neonicotinoids, sulfoxaflor and triflumezopyrim, and one synthetic pyrethroid, cypemethrin, have been evaluated as fire ant bait AIs [12,13,14]. Although all these three insecticides showed promising effect, no bait products based on these insecticides are available in the current US market.

### 2.2. Naturally Occurring Compounds Used in Current Fire Ant Baits

Abamectin and spinosad are the only two bait AIs that are not synthetic insecticides (Table 1). Abamectin and spinosad are fermentation products of bacteria *Streptomyces avermitilis* and *Saccharopolyspora spinosa* respectively [15]. Abamectin is a mixture of avermectins containing primarily avermectin B1a and avermectin B1b and spinosad a mixture of spinosyn A and spinosyn D. Currently there are five commercial fire ant bait products based on these natural compounds (Table 2). Antixx^®^ Fire Ant Bait is the only bait that has been certified for use in organic production by the Organic Materials Review Institute (OMRI)(Eugene, OR, USA) [16].

In addition to organic AIs, boron based inorganic compounds have been tested as fire ant bait AIs, particularly borax and boric acid. These two compounds have a long history of use for controlling ants and their toxicity has been investigated in many ant species, such as Florida carpenter ants, *Camponotus floridanus* (Buckley) [17], Argentine ants, *Linepithema humile* (Mayr) [18,19], *Dolichoderus thoracicus* (Smith), a widespread ant species in Asia [20], and fire ants [21]. Boron compound-based ant bait products are commercially available for controlling many ant species, particularly indoor pest ants. Although fire ants are generally considered an outdoor pest, they occasionally migrate into homes for shelter and food, to escape outdoor harsh conditions, such as extreme heat, drought, or flooding. The boric acid/sucrose water bait has successfully eliminated large laboratory fire ant colonies [22] and there are numerous recipes of boron compound-based homemade fire ant control products found on the internet. However, sufficient scientific data that support their efficacy for controlling fire ants in the field is lacking.

### 2.3. Naturally Occurring Compounds that are Recently Evaluated as Potential Fire Ant Bait Active Ingredients

Effort has been made in searching for naturally occurring compounds as fire ant bait AIs. Five compounds isolated from the root powder of *Periploca sepium* Bunge (Asclepiadaceae), including four pregnane glycosides and one oligasaccharide, possess oral toxicity against fire ants. Among these five compounds, the periplocoside x, a pregnane glycoside, showed the greatest toxicity [23] where it induced a severe, time-dependent cytotoxicity in the midgut epithelial cells of fire ants [24].

Several common natural compounds have been found to be toxic to fire ants by ingestion. It was found that intake of glutamic acid monosodium salt hydrate, glycine, L-alanine, succinic acid, succinic acid disodium, inosinate 5′-monophosphate disodium salt hydrate, or guanosine 5′-monophosphate disodium salt caused mortality of fire ants. Glycine and guanosine 5′-monophosphate disodium salt exhibited the strongest toxicities, causing 100% mortality in workers after 84 h. LC_50_ values were 0.004 g/mL and 0.02 g/mL for guanosine 5′-monophosphate disodium salt and glycine, respectively [25]. It was found that intake of various sweeteners such as erythritol, aspartame, or saccharin caused significant mortality in fire ants [26]. The mortality of the workers could reach above 80% after 72 h feeding on 0.1 or 0.2 g/mL erythritol. The mortality of males, females, and larvae could reach close to 100% after 9 d feeding at high concentrations. The effect of erythritol was found to be dose-dependent for workers, males, females, and larvae. Transfer of erythritol among the fire ant colonies was also observed. Toxicity of aspartame was not observed on other ants, such as black garden ants, *Lasius niger* [27], indicating a possible species selectivity of its toxicity toward ants. The toxicity of erythritol was well documented for many other insect species, such as fruit fly [28], termites [29], house fly and stable fly [30,31], pear psylla [32]. mosquitoes [33], and pavement ant, *Tetramorium immigrans* Santschi [34].

Due to their slow-acting property and low mammalian toxicity, these compounds may be potentially useful as AIs or additives in fire ant baits. However, before these compounds are used in the control of fire ants, it is necessary to have a better understanding on their modes of action. It is also worthy to further conduct structure-activity relationship analyses for these compounds, which may lead to more promising molecules.

## 3. Alternatives to Synthetic Chemical Insecticides for Use in Fire Ant Contact-Based Control Products

### 3.1. Overview

Contact insecticides are commonly used in spray/broadcast/drench/dust products to control fire ants. They are mainly used in area surface and mound treatments. Area surface application of contact insecticides required relatively more insecticides than other methods, and it also has greater environmental impact by eliminating other ant species in the treated areas. The fire ant is a mound-building ant species, where the mound is the above ground portion of the colony’s nest. The mounds of large fire ant colonies are quite visible, particularly when weather is cool and wet, such as in early spring. In the dry and hot summer, fire ants tend to stay deeper in the soil, so mounds may be less visible; however, a rainfall in the dry season will induce ants to rebuild a visible mound. The visibility of the mound makes it possible to kill ants by directly treating the ant nests using fast acting contact AIs. Since the insecticides are applied directly on the mound, its impact on non-target organisms is significantly reduced.

Over 30 AIs are used in various contact insecticide products registered for use against fire ants in the U.S. (Table 3) [35,36,37]. In addition to the synthetic insecticides, a number of naturally occurring organic compounds/mixtures have been used in controlling fire ants, including pyrethrins, spinosad, d-limonene, rotenone, and several plant essential oils. Diatomaceous earth, a natural inorganic material, has been used in treating fire ant mounds. Unlike other AIs that function as chemical toxins, diatomaceous earth physically damages the wax layers on the exoskeleton of insects, which causes them to die by desiccation. Treating fire ant mound with hot water [38], hot air [39] or liquid nitrogen [40] have been used to kill fire ant colonies. Apparently, in all three cases, ants died of physical impact from the lethal temperatures rather than chemical toxicity.

### 3.2. Naturally Occurring Compounds Used in Current Fire Ant Contact-Based Control Products

Pyrethrins were well-known naturally occurring insecticides that were first used around 1800s to control body lice [41]. Pyrethrins are broad-spectrum insecticides and widely used for controlling various pest insects. It is not surprising that they are also effective against fire ants. As insect nerve poisons, pyrethrins can kill ants within minutes to hours. Pyrethrin based products are usually used in ant mound treatments or surface sprays. In some pyrethrin products, piperonyl butoxide (PBO) was used as a synergist. Pyrethrins has been also used together with diatomaceous earth for mound treatments, most likely because diatomaceous earth alone is not effective enough to kill whole fire ant colonies. Pyrethrins have low toxicity to human and other mammals and can break down quickly in the environment, particularly in the presence of sunlight, therefore they are considered among the safest insecticides in the market. Unfortunately, bees and many aquatic organisms are highly sensitive to pyrethrins [42].

Several spinosad based products are available in the market for fire ant mound drench, such as Monterey Garden Insect Spray (Lawn and Garden Products, Inc., Fresno, CA, USA), Ferti-Lome^®^ Borer, Bagworm, Leafminer, and Tent Caterpillar Spray (Voluntary Purchasing Groups, Inc., Bonham, TX, USA), and Entrust^®^ SC Naturalyte^®^ Insect Control (Dow AgroScience LLC, Indianapolis, IN, USA). These products are primarily designed for controlling lepidopteran pests in home gardens and landscapes, but they can be used as mound drenches for fire ants. Both Monterey Garden Insect Spray and Entrust^®^ SC Naturalyte^®^ Insect Control are OMRI listed [16]. It is noteworthy that spinosad is among the few active ingredients in both baits and a contact insecticide in granules and liquid formulations for mound and area treatments. Rotenone and fipronil are the only other active ingredients that have been used for both types of fire ant control products.

Rotenone is a compound produced in the roots or rhizomes of the tropical legumes *Derris*, *Lonchocarpus*, and *Tephrosia*, which has been used as an insecticide for 165 years. It interferes with the electron transport chain in mitochondria and prevents energy production [43]. Prior to the discovery and commercialization of spinosad, rotenone had been used as a broad-spectrum insecticide for the control of various pest insects, including fire ants. However, use of rotenone has been phased out in the USA since it was found that acute exposure to rotenone in rats produced brain lesions consistent with those observed in humans and animals with Parkinson’s disease, and that rotenone residue was persistent on some crops after treatment [44]. However, research on using rotenone and rotenone containing extract for controlling ants is still active in other countries, such as China. For example, extracts of *Tephrosia vogelii* and *Derris trifoliata*, and root powder of *Derris hancei* were evaluated as bait AIs against fire ants under both laboratory and field conditions [45,46]. Rotenone serves as a good example that not all the natural products are necessarily safer than synthetic insecticides.

Plant essential oils and extracts have been used for controlling fire ants as mound treatment products. For example, Orange Guard Fire Ant Control (Orange Guard, Inc., Marina, CA, USA) is a product based on citrus peel extract (5.8% d-limonene) and Essentria^®^ G Granular Insecticide (former EcoEXEMPT™ G Granular Insecticide) is based on eugenol (2.9%) and thyme oil (0.6%) (Central Garden & Pet Company, Schaumburg, IL, USA). Essentria^®^ IC-3 Insecticide Concentrate is based on rosemary oil (10%), geraniol (5%) and peppermint oil (2%) (Central Garden & Pet Company, Schaumburg, IL, USA). Three experimental citrus oil-based formulations were tested in the field [47]. Since d-limonene and eugenol are the dominant component in citrus peel extracts and clove oil respectively, d-limonene or eugenol are commonly listed as the active ingredients on product labels. However, many minor compounds were identified in these oils/extracts. For example, 54 components were identified in the steam distilled essential oils and 44 in the extracts of orange peels of 12 cultivars of *Citrus sinensis* from central-eastern Sicily, Italy [48]. Although in all the cultivars, the main component is d-limonene (73.9–97%), numerous monoterpenes and terpenoids were also found. A total of 23 constituents were identified in clove essential oil [49]. In thyme essential oil, 56 components were identified, and the oxygenated monoterpenes were the most abundant (57.14%) and dominated by geraniol (22.33%), geranyl acetate (19.38%), and linalool (5.49%). Thymol is a dominate phenolic compound in thyme oil (14%) [50]. Thirty-four compounds were tentatively identified in rosemary essential oil, with α-pinene, bornyl acetate, camphene and 1,8-cineole being the major components [51]. Components of peppermint leaf essential oil include menthol, menthone, menthofuran, 1,8-cineole, and menthyl acetate [52]. Minor compounds can play a significant role. For example, LC_50_ and LC_90_ values of clove essential oil against mosquitoes, *Anopheles stephensi* were significantly lower than those of eugenol, indicating other minor compounds contribute significantly to the toxicity of clove oil [53]. Although essential oil-based control products are available in the market, the data on toxicity of these essential oils and their individual component to fire ant are generally lacking; especially, the contribution of minor compounds to the toxicity of essential oils has not been well studied. Essential oils are produced in over 17,500 plant species [54]. They may become an important source of naturally occurring compounds/mixture that have a potential to be used in fire ant management.

### 3.3. Naturally Occurring Organic Compounds/Materials that Have Been Evaluated as Contact-Based Control Toxins

Many naturally occurring organic compounds/materials have been assessed for their contact toxicity against fire ants (Table 4), including defensive compounds from other ants [55,56,57], anuran skin alkaloids [58], plant raw materials, plant extracts and essential oils and their individual components [45,59,60,61,62,63,64,65,66,67].

Several compounds and materials have been evaluated in the field and results are encouraging. For example, in a mound drench test essential oil of the Alaskan yellow cedar, *Cupressus nootkatensis* (D. Don) Spach, caused higher mortality of hybrid imported fire ant mounds (*Solenopsis invicta* Buren × *Solenopsis richteri* Forel) than the untreated check at the end of the 12-wk evaluation period. In a band application evaluation, plots treated with this essential oil maintained a 90–95% reduction in fire ant mounds from the 2nd to 17th wk [59].

2-tridecanone is a major constituent of defensive secretions in tawny crazy ants, *Nylanderia fulva*, an ant species that was reported to be able to displace red imported fire ants in the field [56]. 2-tridecanone is also a major part of the *N. fulva* synergistic pheromone system [72]. Two 2-tridecanone-based emulsifiable concentrates (in one formulation, piperonyl butoxide used as a synergist) were evaluated in the field as mound drench treatments [73]. At application rate of 5.28 mL/L and 14 days after mound drench treatment, 100% control was achieved with the PBO formulation and 90% control with the formulation without PBO. Due to low mammalian toxicity, no presence of hazardous synthetic organic solvents, no phytotoxicity at applied concentrations, and relatively low cost, both formulations of 2-tridecanone are promising alternatives to commercial insecticide products for fire ant mound drenches.

Among 15 commercially available benzoates, benzylbenzoate, n-pentybenzoate, and hexylbenzoate showed strongest contact toxicity against fire ants [68]. Hexylbenzoate is a naturally occurring compound, which is present in apricot, lingonberry, cowberry, feijoa, peach, sapodilla, Parmesan cheese, butter and black tea. Hexyl benzoate is used as a flavoring ingredient (PubChem CID: 23235, URL: https://pubchem.ncbi.nlm.nih.gov/compound/Hexyl-benzoate). Since hexylbenzoate does not have Occupational Safety and Health Administration hazards and its risk to the environment is acceptable [74], it has been formulated as an emulsifiable concentrate and the evaluation of this formulation as a mound drench treatment is ongoing (Chen, unpublished data).

## 4. Fire Ant Repellants

### 4.1. Overview

Fire ants are very aggressive after mound disturbance. When a person steps into a fire ant mound, probably no repellant can deter the agitated fire ants from stinging the intruder [75]. However, repellants can be useful in many other scenarios. They can be used to prevent fire ants from invading sensitive areas, such as electrical equipment, nursing homes, and hospitals. They are also potentially useful in suppressing the spread of fire ants by preventing them from re-entering quarantined objects/materials such as nursery stock and soil-moving equipment. Some synthetic insecticides can function as fire ant repellants. For example, bifenthrin and tefluthrin were used to repel fire ants from potting soil [76] and permethrin-impregnated nylon plastics for repelling fire ants from electrical housings and equipment boards [77]. Tremendous effort has been made in searching for effective and safe repellant alternatives, with numerous compounds/materials being investigated. Many classes of organic compounds have been reported as fire ant repellants (Table 5), such as carboxylic acids, alcohols, carboxylic and dicarboxylic acid esters, ketones [78,79], phthalates [80], terpenes, terpenoids [67,81], phenylpropanoids [64], allylbenzes [63], aminobenzenes [82], alkylamines [57], alkylpyridine (Zhou et al. unpublished data), and pyrone derivative [83].

### 4.2. Naturally Occurring Organic Compounds/Materials that Have Been Evaluated as Fire Ant Repellants

The repellency of many naturally occurring compounds/materials has been tested against fire ants (Table 5), including defensive compounds from other ants [57,84], plant raw materials [62,64], plant essential oils and their individual components [59,60,67,81,85,86,87,88,90,91,92,94,98].

Many plant essential oils exhibit repellency against fire ants, such as ylang ylang oil (Du et al. unpublished data), nootka oil [59], mint oil [60], and essential oils of *Salvia sclarea* L., *Capsicum annuum* L., *Mentha canadensis* L., *Mentha longifolia* (L.) Huds., *Cedrus deodara* (Roxb.) G.Don, *Pinus* spp. [90], *Eucalyptus globulus* Labill, *Artemisia carvifolia* Buch.-Ham. ex Roxb [94], *Cymbopogon nardus* (L.) Rendle, *Cinnamomum cassia* (L.) J.Pres, and *Ilex purpurea* Hassk [93]. A Chinese essential oil product also show repellency against fire ants [81,89,90]. There are about 17,500 higher plant species that produce essential oils [54], but only small fraction of essential oils has been tested on fire ants, indicating that plant essential oils may be a rich source of new fire ant repellants.

Fire ant repellants are usually compounds with low molecular weight, so they are very volatile. The application of those compounds under field conditions may require improved delivery technologies in order to achieve a sustained efficacy. Recently, nanoparticle encapsulation technique has been used to formulate essential oils for pest insect control [99]. A nanoformulation of essential oil of *Pogostemon cablin* (Blanco) Benth for controlling leaf-cutting ants, *Atta opaciceps* (Borgmeier), *Atta sexdens* (Linnaeus), *Atta sexdens rubropilosa* Forel has been reported [100].

One noteworthy factor is that some compounds/materials showed opposite biological effects based on concentrations. Ylang ylang oil exhibited repellency against fire ants at high concentrations, but attractancy at low concentration (Du et al. unpublished data). Similar phenomenon was observed for individual compounds, such as eucalyptol [81], prenyl acetate and pentyl acetate (Du et al. unpublished data). In the field, after the concentration of a repellant decreases with time, it may become an attractant, entirely opposite to its intended effect. Such materials and compounds may not be suitable to be used as fire ant repellants.

## 5. Fire Ant Fumigants

### 5.1. Overview

Fire ants build earthen nests that are relatively closed system, which makes nest fumigation a possible treatment option in fire ant management. A number of insecticides have been tested as fire ant fumigants [7,101,102]. 1,1,1,trichloroethane, an organic solvent, was used as fumigant for treating fire ant mounds [103]. Since 1,1,1-trichloroethane is an ozone-depleting substance, its use is being rapidly phased out. Although positive results were observed in some cases, fumigation is not well exploited in managing fire ants. To our knowledge, there are only two fumigant-based products available in the current US market: Ant-Zap Ant Killer Mound Destroyer (Trifecta, LLC, Fayetteville, Arkansas) and Earthfire Injection System (Earthfire Fire Ant Eradication, Scottsdale, Arizona). Ant-Zap Ant Killer Mound Destroyer is CO_2_ based fumigant product for fire ant mound treatment. Although this product is included in the OMRI list, scientific data to support its effectiveness is not available. Earthfire Injection System uses heat to vaporize the insecticide resmethrin and inject the toxic vapor into fire ant nests. This system caused a 84% reduction of fire ant mound activity in a field trial [7].

Fumigants may be useful in fire ant quarantine treatments. Fire ants are a major concern for many countries facing the risk of its invasion. Eradication of ant colonies in the early stage of the invasion is key to prevent the new establishment of fire ants. In South Korea, there are multiple recent detections of fire ants and majority of those detections were around the main port areas of imported common trade containers, which indicates that disinfestation of the containers is necessary to prevent the introduction of fire ants. Ethyl formate was found to be effective in controlling 99.9% of fire workers and female alates at 46.1 and 37.7 g h m^−3^ (lethal concentration × time) at 13 °C and 23 °C respectively, which provided strong support to further explore its use as a fumigant to disinfest fire ants from commerce shipping containers [104].

Fire ants were first detected in Japan in a shipping container from China in May 2017. Allyl isothiocyanate, a naturally occurring compound, is a potent fumigant against fire ants [61]. Fumigation using microencapsulated allyl isothiocyanate pellets in a gas-barrier bag was tested in Japan [105]. Fire ants were completely killed within 24 h. Allyl isothiocyanate, a compound found in wasabi, *Eutrema japonicum* (Miq.) Koidz, is safe for humans and the environment; however, its use as fumigant has been limited by its high volatility and strong pungency. Encapsulation seems a solution to this problem [105].

### 5.2. Naturally Occurring Compounds/Materials that Have Been Evaluated as Fire Ant Fumigants

In addition to ethyl format and allyl isothiocyanate, many other naturally occurring compounds/materials have been tested as fumigants against fire ants (Table 6), including defensive compounds from other ants [55], plant materials [106,107,108], plant essential oils and their individual components [59,66,67,68,86,94,109,110]. As expected, some fire ant repellants are also efficient fire ant fumigants. Many plant essential oils are fumigants against fire ants, such as sweet wormwood oil [67], *Artemisia annua* oil, eucalyptus oil, wintergreen oil, mugwort oil, chrysanthemum oil, turpentine oil, forsythia oil [109], and sweet orange oil [110]. Plant essential oils may also be a rich source of new fire ant fumigants.

Several compounds are well worth further investigation. Ethyl formate is a volatile compound which occurs naturally in a variety of products, including beef, cheese, rice, grapes and wine, and is generally recognized as a safe (GRAS) compound. As a methyl bromide alternative, it has been used as a fumigant of dried fruit and is under investigation as a fumigant for other commodities. Methyl benzoate is another GRAS compound approved by the FDA for human administration. The Council of Europe has listed methyl benzoate in the table of artificial flavors which can be used for food and is harmless to human health with the maximum amount of 61 mg/kg and ADI being 5 mg/kg. In addition to being used in quarantine treatments for disinfesting containers, these compounds may be also useful in treating fire ant mounds.

## 6. Conclusions

Naturally occurring compounds/materials have been successfully used as active ingredients in fire ant baits and contact-based control products. Numerous fire ant repellants and fumigants have also been identified. However, these compounds/materials may represent only a small fraction of such compounds available in the nature. More rigorous and extensive research on this topic is expected and more collaboration with the insecticide industry is certainly needed to commercialize the identified active compounds/materials.

## Figures and Tables

**Table 1:** Active ingredients currently used in commercial fire ant baits in the USA.

Active Ingredient	Source	Mode of Action
Abamectin	Natural compounds from bacterium *Streptomyces avermitilis*	Selective high-affinity binder to glutamate-gated chloride channels
Fipronil	Synthetic phenylpyrazole	Blocker of gamma-aminobutyric acid (GABA)-gated chloride channels and glutamate-gated chloride channels
Hydramethylnon	Synthetic trifluoromethyl aminohydrazone	Metabolic inhibitor.
Indoxacarb	Synthetic oxadiazine	Blocker of neuronal sodium channels
Metaflumizone	Synthetic semicarbazone	Blocker of neuronal sodium channels
Methoprene	Synthetic juvenile hormone analog	Insect growth regulator
Pyriproxyfen	Synthetic juvenile hormone analog	Insect growth regulator
Spinosad	Natural compounds from bacterium *Saccharopolyspora spinosa*	Disruptor of acetylcholine neurotransmission

**Table 2:** Fire ant bait products that use Abamectin or Spinosad as active ingredients.

Product Name	AI and Concentration	Company
Clinch	abamectin (0.011%)	Syngenta Crop Protection. LLC, Greensboro, NC, USA
Ferti-lome Come and Get It Fire Ant Killer	spinosad (0.015%)	Voluntary Purchasing Group, Bonham, TX, USA
Payback Fire Ant Bait	spinosad (0.015%)	Southern Agricultural Insecticides, Inc. (Palmetto, FL; Hendersonville, NC; Boone, NC, USA
Antixx^®^ Fire Ant Bait	spinosad (0.015%)	W. Neudorff GmbH KG, Emmerthal, Germany

**Table 3:** Active ingredients used in contact insecticide products for fire ant control.

Active Ingredient	Type	Mode of Action
Pyrethrins	Natural compound in *Chrysanthemum cinerariifolium*	Interfere with sodium channel gating
d-Limonene	Natural compound in oil of citrus fruit peels	Not clearly understood, most likely with multiple effects
Rotenone	Natural compound in seeds and stems of several plants	Interfere with the electron transport chain in mitochondria
Spinosad	Natural compounds in bacterium Saccharopolyspora spinosa	Disrupt acetylcholine neurotransmission
Clove oil	Natural essential oil	Not clearly understood, most likely with multiple effects
Cotton seed oil	Natural essential oil	Not clearly understood, most likely with multiple effects
Lemongrass oil	Natural essential oil	Not clearly understood, most likely with multiple effects
Peppermint oil	Natural essential oil	Not clearly understood, most likely with multiple effects
Pine oil	Natural essential oil	Not clearly understood, most likely with multiple effects
Rosemary oil	Natural essential oil	Not clearly understood, most likely with multiple effects
Turpentine	Natural essential oil	Not clearly understood, most likely with multiple effects
Carbaryl	Synthetic carbamate	Inhibit acetylcholinesterase
Imidacloprid	Synthetic neonicotinoid	Bind to nicotinic acetylcholine receptors and trigger cell responses
Acephate	Synthetic organophosphates	Inhibit acetylcholinesterase
Fipronil	Synthetic phenylpyrazole	Block GABA-gated chloride channels and glutamate-gated chloride channels
Allethrin	Synthetic pyrethroid	Interfere with sodium channel gating
beta-Cyfluthrin	Synthetic pyrethroid	Interfere with sodium channel gating
Bifenthrin	Synthetic pyrethroid	Interfere with sodium channel gating
Cypermethrin	Synthetic pyrethroid	Interfere with sodium channel gating
Deltamethrin	Synthetic pyrethroid	Interfere with sodium channel gating
es-Fenvalerate	Synthetic pyrethroid	Interfere with sodium channel gating
Fenvalerate	Synthetic pyrethroid	Interfere with sodium channel gating
Fluvalinate	Synthetic pyrethroid	Interfere with sodium channel gating
Lambda-Cyhalothrin	Synthetic pyrethroid	Interfere with sodium channel gating
Permethrin	Synthetic pyrethroid	Interfere with sodium channel gating
Resmethrin	Synthetic pyrethroid	Interfere with sodium channel gating
s-Bioallethrin	Synthetic pyrethroid	Interfere with sodium channel gating
Sumethrin	Synthetic pyrethroid	Interfere with sodium channel gating
Tefluthrin	Synthetic pyrethroid	Interfere with sodium channel gating
Tetramethrin	Synthetic pyrethroid	Interfere with sodium channel gating
Tralomethrin	Synthetic pyrethroid	Interfere with sodium channel gating

**Table 4:** Naturally occurring organic compounds/materials that have been evaluated as contact-based toxins *.

Compound	References
Formic acid	[55,56]
Methyl benzoate analogs	[68]
Clove powder	[63]
Eugenol	[63]
Eugenol acetate	[63]
Beta-caryophyllene	[63]
Sweet wormwood	[67]
Camphor	[67]
Linalool	[67]
Cineole	[67]
α-Terpineol	[67]
L-(-)-Borneol	[67]
Decylamine	[57]
Dodecylamine	[57]
Mint oil	[60]
2-Tridecanone	[56]
Undecane	[56]
Batrachotoxinin A	[58]
Pumiliotoxin 237A **	[58]
(5E, 9E) 3-Butyl-5-propylindolizidine 223AB	[58]
Decahydroquinoline cis-223F	[58]
Pseudophrynaminol	[58]
Spiropyrrolizidine oxime	[58]
(+)-Gephyrotoxin	[58]
5,8-Disubstituted indolizidine 205A	[58]
5,8-Disubstituted indolizidine 235B	[58]
Octahydrohistrionicotoxin 291A	[58]
2-Methyl-6-undecylpiperidine	[58]
Pumiliotoxin 267C	[58]
Batrachotoxin	[58]
Histrionicotoxin	[58]
Nicotine	[58]
Pumiliotoxin 323A	[58]
Pumiliotoxin 251D	[58]
Allopumiliotoxin 267A	[58]
Pumiliotoxin 307A	[58]
Batrachotoxin	[58]
Octahydrohistrionicotoxin 291A	[58]
Allodihydrohistrionicotoxin 285A	[58]
Histrionicotoxin 259A	[58]
Pumiliotoxin 267C	[58]
Pumiliotoxin 251D	[58]
Cinnamon leaf essential oil	[69]
*trans*-cinnamaldehyde	[69]
allyl isothiocyanate	[61]
3-butenyl isothiocyanate	[61]
3-(methylthio)propyl isothiocyanate	[61]
2-phenylethyl isothiocyanate	[61]
Cupressus nootkatensis essential oil	[59]
*Acorus calamus* powder	[64]
α-Asarone	[64]
α-Asarone	[64]
Soil in the rhizosphere of *Viburnum odoratissimum*	[70]
Soil containing cinnamon leaf debris	[62]

*: In some studies, the contact and fumigation toxicities of test compounds/materials were not well separated due to the experimental design. Those compounds/materials were not included in the table. For example, in studies that examined the biological activity of essential oils of *Cinnamomum camphora, Cinnamomum glaucescens*, *Cinnamomum tamala*, *Amomum subulatum*, and *Aegle mamelos* from Nepal, a test compound/material was sprayed onto a filter paper at the bottom of a beaker, and fire ant workers were transferred into the beaker, which was then sealed with parafilm. The mortality of fire ants was recorded after 24 h. Apparently, both contact and fumigation toxicities contributed to the observed mortality [65,66,71]. **: The number after name is molecular weight and the letter after number is used to differentiate isomers.

**Table 5:** Compounds/materials that have been evaluated as fire ant repellants.

Compound/Material	References
Formic acid	[84]
Clove powder	[63]
Eugenol	[63]
Eugenol acetate	[63]
Beta-caryophyllene	[63]
Sweet wormwood oil	[67]
Camphor	[67]
Linalool	[67]
Cineole	[67,85]
α-Terpineol	[67,85]
L-(-)-Borneol	[67]
Decylamine	[57]
Dodecylamine	[57]
D-Limonene	[85]
Mint oil	[60]
Camphor oil	[86]
*Cupressus nootkatensis* essential oil	[59]
*Acorus calamus* powder	[64]
α-Asarone	[64]
β-Asarone	[64]
Soil in rhizosphere of *Viburnum odoratissimum*	[70]
Soil containing cinnamon leaf debris	[62]
Anthranilate	[82]
Butyl anthranilate	[82]
Callicarpenal	[87]
Intermedeol	[87]
*Hedychium* essential oil	[88]
Feng Yu Jing, a Chinese essential oil product	[81,89,90]
Viridiflorol	[85]
*E*-Nerolidol	[91]
α-Pinene	[85]
β-Pinene	[85]
α-Terpinene	[85]
γ-Terpinene	[85]
Terpinen-4-ol	[85]
*E*-Nerolidol	[91]
β-Caryophyllene	[91]
Vetiver oil	[92]
*Salvia sclarea* oil	[93]
*Capsicum annuum* oil	[93]
*Cymbopogon nardus* oil	[93]
*Ilex purpurea* oil	[93]
*Cinnamomum cassia* oil	[93]
*Mentha Canadensis* oil	[90]
*Salvia sclarea* oil	[90]
*Capsicum annuum* oil	[90]
*Mentha longifolia oil*	[90]
*Pinus* spp oil	[90]
*Cedrus deodara* oil	[90]
*Eucalyptus globulus* essential oil	[94]
*Artemisia carvifolia* essential oil	[94]
Sage (Saliva sp.) shaving water suspension	[95]
Pine needle shaving water suspension	[95]
Cedar shaving water suspension	[95]
Grass WW-B.Dahl	[96]
Red cedar mulch	[97]
Pine bark mulch	[97]
Red cypress mulch	[97]
Hardwood mulch	[97]
Basil essential oil	[98]
Citronella essential oil	[98]
Lemon essential oil	[98]
Peppermint essential oil	[98]
Tea tree essential oil	[98]
Eucalyptus essential oil	[98]
Carboxylic acids	[78]
Carboxylic acid and dicarboxylic acid esters	[78,79]
Alcohols	[78]
Ketones	[78]
Ylang ylang oil	(Du et al. unpublished data)
Sodium dehydroacetate	[83]

**Table 6:** Compounds/materials that have been evaluated as fire ant fumigants.

Compound	References
Formic acid	[55,56]
Methyl benzoate analogs	[68]
Sweet wormwood	[67]
d-Camphor	[67]
Linalool	[67]
Cineole	[67]
α-terpineol	[67]
L-(-)-borneol	[67]
Camphor oil	[109]
*Artemisia annua* oil	[109]
Eucalyptus oil	[109]
Wintergreen oil	[109]
Mugwort oil	[109]
Chrysanthemum oil	[109]
Turpentine oil	[109]
Forsythia oil	[109]
Sweet orange oil	[110]
d-limonene	[110]
Linalool	[110]
Heptanone	[111]
Octanone	[111]
Nonanone	[111]
Undecanone	[111]
2-Tridecanone	[56]
Undecane	[56]
Camphor oil	[86]
Camphor	[86]
Cineole	[86]
Ethyl formate	[104]
Allyl isothiocyanate	[61,105]
3-Butenyl isothiocyanate	[61]
3-(Methylthio)propyl isothiocyanate	[61]
2-Phenylethyl isothiocyanate	[61]
*Cupressus nootkatensis* essential oil	[59]
*Michelia alba* leaves	[106]
*Murraya exotica* leaves	[107]
*Tephrosia vogelii* fresh material	[108]
*Eucalyptus globulus* essential oil	[94]
*Artemisia carvifolia* essential oil	[94]
